# Ranking-Based Convolutional Neural Network Models for Peptide-MHC Class I Binding Prediction

**DOI:** 10.3389/fmolb.2021.634836

**Published:** 2021-05-17

**Authors:** Ziqi Chen, Martin Renqiang Min, Xia Ning

**Affiliations:** ^1^ Computer Science and Engineering Department, The Ohio State University, Columbus, OH, United States; ^2^ Machine Learning Department, NEC Labs America, Princeton, NJ, United States; ^3^ Biomedical Informatics Department, The Ohio State University, Columbus, OH, United States; ^4^ Translational Data Analytics Institute, The Ohio State University, Columbus, OH, United States

**Keywords:** deep learning, prioritization, peptide vaccine design, convolutional neural networks, attention

## Abstract

T-cell receptors can recognize foreign peptides bound to major histocompatibility complex (MHC) class-I proteins, and thus trigger the adaptive immune response. Therefore, identifying peptides that can bind to MHC class-I molecules plays a vital role in the design of peptide vaccines. Many computational methods, for example, the state-of-the-art allele-specific method MHCflurry, have been developed to predict the binding affinities between peptides and MHC molecules. In this manuscript, we develop two allele-specific Convolutional Neural Network-based methods named ConvM and SpConvM to tackle the binding prediction problem. Specifically, we formulate the problem as to optimize the rankings of peptide-MHC bindings via ranking-based learning objectives. Such optimization is more robust and tolerant to the measurement inaccuracy of binding affinities, and therefore enables more accurate prioritization of binding peptides. In addition, we develop a new position encoding method in ConvM and SpConvM to better identify the most important amino acids for the binding events. We conduct a comprehensive set of experiments using the latest Immune Epitope Database (IEDB) datasets. Our experimental results demonstrate that our models significantly outperform the state-of-the-art methods including MHCflurry with an average percentage improvement of 6.70% on AUC and 17.10% on ROC5 across 128 alleles.

## 1 Introduction

Immunotherapy, an important treatment of cancers, treats the disease by boosting patients’ immune systems to kill cancer cells (Mellman et al., 2011; Couzin-Frankel, 2013; Esfahani et al., 2020; Waldman et al., 2020). To trigger patients’ adaptive immune responses, Cytotoxic T cells, also known as CD8^+^ T-cells, have to recognize peptides presented on the cancer cell surface (Valitutti et al., 1995; Blum et al., 2013). These peptides are fragments derived from self-proteins or pathogens by proteasomal proteolysis within the cell. To have the peptides presented on the cell surface to be recognized by CD8 receptors, they need to be brought from inside the cells to the cell surface, typically through binding with and transported by major histocompatibility complex (MHC) class-I molecules. To mimic natural occurring proteins from pathogens, synthetic peptide vaccines are developed for therapeutic purposes (Purcell et al., 2007). Therefore, to design successful peptide vaccines, it is critical to identify and study peptides that can bind with MHC class-I molecules.

Many computational methods have been developed to predict the binding affinities between peptides and MHC class-I molecules (Han and Kim, 2017; O’Donnell et al., 2018). These existing computational methods can be categorized into two types: allele-specific methods and pan methods. Allele-specific methods train one model for one allele such that the model can capture binding patterns specific to the allele, and thus it is better customized to that allele (Lundegaard et al., 2008; O’Donnell et al., 2018). Pan methods train one model for all the alleles at the same time, and thus the information across different alleles can be shared and integrated into a general model (Jurtz et al., 2017; Hu et al., 2018). These existing methods can achieve significant performance on the prediction of binding affinities. However, most existing methods formulate the prediction problem as to predict the exact binding affinity values (e.g., IC_50_ values) via regression. Such formulations may suffer from two potential issues. First of all, they tend to be sensitive to the measurement errors when the measured IC_50_ values are not accurate. In addition, many of these methods use ranking-based measurement such as Kendall’s Tau correlations to measure the performance of regression-based methods (Bhattacharya et al., 2017; O’Donnell et al., 2020). This could lead to sub-optimal solution as small regression errors do not necessarily correlate to large Kendall’s Tau. Therefore, these methods are limited in their capability of prioritizing the most possible peptide-MHC pairs of high binding affinities.

In this study, we formulate the problem as to prioritize the most possible peptide-MHC binding pairs via ranking based learning. We propose three ranking-based learning objectives such that through optimizing these objectives, we impose peptide-MHC pairs of high binding affinities ranked higher than those of low binding affinities. Coupled with these objectives, we develop two allele-specific Convolutional Neural Network (CNN)-based methods with attention mechanism, denoted as ConvM and SpConvM. ConvM extracts local features of peptide sequences using 1D convolutional layers, and learns the importance of different positions in peptides using self-attention mechanism. In addition to the local features used in ConvM, SpConvM represents the peptide sequences at different granularity levels by leveraging both global and local features of peptide sequences. We also develop a new position encoding method together with self-attention mechanism so as to differentiate amino acids at different positions. We compare the various combinations of model architectures and objective functions of our methods with the state-of-the-art baseline MHCflurry (O’Donnell et al., 2018) on IEDB datasets (Vita et al., 2018). Our experimental results demonstrate that our models significantly outperform the state-of-the-art methods with an average percentage improvement of 6.70% on AUC and 17.10% on ROC5 across 128 alleles.

We summarize our contributions below:

- We formulate the problem as to optimize the rankings of peptide-MHC pairs instead of predicting the exact binding affinity values. Our experimental results demonstrate that our ranking-based learning is able to significantly improve the performance of identifying the most possible peptide-MHC binding pairs.
- We develop two allele-specific methods ConvM and SpConvM with position encoding and self attention, which enable a better learning of the importance of amino acids at different positions in determining peptide-MHC binding.
- We incorporate both global and local features in SpConvM to better capture and learn from different granularities of peptide sequence information.
- Our methods outperform the state-of-the-art baseline MHCflurry on IEDB datasets (O’Donnell et al., 2018) in prioritizing the most possible peptide-MHC binding pairs.

## 2 Literature Review

The existing computational methods for peptide-MHC binding prediction can be generally classified into two categories: linear regression-based methods and deep learning (DL)-based methods. Below, we present a literature review for each of the categories, including the key ideas and the representative work.

### 2.1 Peptide Binding Prediction Via Linear Regression

Many early developed methods on peptide-MHC binding prediction are based on linear regression. For example, Peters and Sette (2005) proposed a method named Stabilized Matrix Method (SMM), which applied linear regression to predict the binding affinities from one-hot encoded vector representation of peptide sequences. Kim et al. (2009) derived a novel amino acid similarity matrix named Peptide:MHC Binding Energy Covariance (PMBEC) matrix and incorporated it into the SMM approach to improve the performance of SMM. In PMBEC, each amino acid is represented by its covariance of relative binding energy contributions with all other amino acids. Some recent work (Zhao and Sher, 2018; Bonsack et al., 2019) demonstrates these linear regression-based methods are inferior to DL-based methods, and therefore, in our work, we focus on DL-based methods.

### 2.2 Peptide Binding Prediction Via Deep Learning

The DL-based models can be categorized into allele-specific methods and pan methods. Allele-specific methods train a model for each allele and learn the binding patterns of each allele separately. Instead, pan methods train a model for all alleles to learn all the binding patterns together within one model. Both the methods use similar encoding methods such Onehot encoding, BLOSUM encoding and Word2Vec (Goldberg and Levy, 2014).

#### 2.2.1 Allele-specific Deep Learning Methods

Among these allele-specific methods, Lundegaard et al. (2008) proposed NetMHC3.0 that takes the embeddings of peptide sequences as input, and they applied neural networks with one hidden layer to predict peptide-MHC binding for peptides of fixed length. In NetMHC3.0, the hidden layer is a fully-connected (FC) layer, and learns the global features of peptide sequences such as the position and types of specific amino acids. Andreatta and Nielsen (2015) extended NetMHC3.0 to NetMHC4.0 by padding so that the model can handle peptides of variable length. Kuksa et al. (2015) developed two nonlinear high-order methods including high-order neural networks (HONN) pre-trained with high-order semi-restricted Boltzmann machine (RBM), and high-order kernel support vector machines (hkSVM). Both the high-order RBMs and the high-order kernel are designed to capture the direct strong high-order interactions between features. Bhattacharya et al. (2017) developed a deep recurrent neural network based on gated recurrent units (GRUs) to capture the sequential features from peptides of various length. Vang and Xie (2017) applied two layers of 1D convolution on the embeddings of peptide sequences so as to learn local binding patterns existing in each *k*-mer amino acids. O’Donnell et al. (2018) designed a deep model named MHCflurry with locally-connected layers. This locally-connected layer is used to learn the position-specific local features from the peptide sequences. MHCflurry has been demonstrated to achieve better or similar performance compared with most of the other prediction methods (Zhao and Sher, 2018; Michael Boehm et al., 2019).

#### 2.2.2 Pan Deep Learning Methods

Nielsen and Andreatta (2016) developed a DL-based pan method named NetMHCpan3.0. This method takes the embedding of pseudo MHC sequences and peptide sequences as input, and then applies an ensemble of neural networks to predict the binding affinities of peptide-MHC pairs. Jurtz et al. (2017) extended NetMHCpan3.0 to NetMHCpan4.0 by training the model on both binding affinity data and eluted ligand data. Their model shares a hidden layer among two kinds of data and applies two different output layers to predict binding affinities and eluted ligands, respectively, for peptide-MHC pairs. Phloyphisut et al. (2019) developed a deep learning model, which uses GRUs to learn the embeddings of peptides, and a FC layer to learn the embeddings of alleles. The two types of embeddings are then concatenated to predict peptide-MHC binding probabilities. Han and Kim (2017) encoded peptide-MHC pairs into image-like array (ILA) data and applied deep 2D convolutional neural networks to extract the possible peptide-MHC interactions from the ILA data. Hu et al. (2018) combined a deep convolutional neural network with an attention module. They applied multiple convolutional layers to extract features of different levels. The extracted features are integrated with the features learned from attention mechanism and fed MHCflurry2.0 into the output layer to predict binding affinities of peptide-MHC pairs. O’Donnell et al. (2020) developed a pan-allele binding affinity predictor BP and an allele-independent antigen presentation predictor MHCflurry2.0 AP to calculate the presentation scores of peptide-MHC pairs. Their binding affinity predictor includes upstream and downstream residues of peptides from their source proteins to improve the performance of models. Note that MHCflurry2.0 is a pan method and requires source proteins of peptides. Therefore, we do not compare our methods with MHCflurry2.0. Venkatesh et al. (2020) developed a model named MHCAttnet which combines a bidirectional long short-term memory (Bi-LSTM) network with attention mechanism to encode the allele sequences and peptide sequences. The encoded peptide embeddings and allele embeddings are then concatenated and fed into the output layer to predict the binding probability. Zeng and Gifford (2019) developed an ensemble of deep residue convolutional neural networks named PUFFIN to predict the probability that the peptide binds to an MHC molecule, and to quantify the uncertainty of binding predictions. Each network in PUFFIN predicts an affinity distribution (i.e., mean and variance) of a peptide-MHC pair. Then, all the predicted mean values and variance values are averaged to produce a final prediction and the uncertainty of the prediction.

## 3 Materials

### 3.1 Peptide-MHC Binding Data

The dataset is collected from the Immune Epitope Database (IEDB) (Vita et al., 2018). Each peptide-MHC entry *m* in the dataset measures the binding affinity between a peptide and an allele. These binding affinity entries could be of either quantitative values (e.g., IC_50_) or qualitative levels indicating levels of binding strength. The mapping between quantitative values and qualitative levels is shown in Table 1. Note that higher IC_50_ values indicate lower binding affinities.

**TABLE 1:** Binding affinity measurement mapping.

**Qualitative**	**Quantitative (nM)**	**Level**
Negative	>5,000	1
Positive-low	1,000–5,000	2
Positive-intermediate	500–1,000	3
Positive	100–500	4
Positive-high	0–100	5

We combined the widely used IEDB benchmark dataset curated by Kim et al. (2014) and the latest data added to IEDB (downloaded from the IEDB website on Jun. 24, 2019). The benchmark dataset contains two datasets BD2009 and BD2013 compiled in 2009 and 2013, respectively. BD2009 consists of 137,654 entries, and BD2013 consists of 179,692 entries. The latest dataset consists of 189,063 peptide-MHC entries. Specifically, we excluded those entries with non-specific, mutant or unparseable allele names such as HLA-A2. We then combined the datasets by processing the duplicated entries and entries with conflicting affinities as follows. We first mapped the quantitative values of all these duplicated or conflicting entries into qualitative levels based on Table 1, and used majority voting to identify the major binding level of the peptide-MHC pairs. If such binding levels cannot be identified, we simply removed all the conflicting entires; otherwise, we assigned the average quantitative values in the identified major binding level to the peptide-MHC pairs. The combined dataset consists of 202,510 entries across 128 alleles and 53,253 peptides as in Table 2. We further normalized the binding affinity values ranging from 0 to 10^7^ to [0, 1] via formula

$$b=\text{clamp}\left(1-\log_{50,000}\left(x\right),0,1\right),$$ (1)

where *x* is the measured binding affinity value, and $\mathrm{clamp}\left(1-\log_{50,000}\left(x\right),0,1\right)$ represents that $1-\log_{50,000}\left(x\right)$ is clamped into range [0, 1]. By using the above clamp function, smaller/larger binding affinity values corresponding to higher/lower binding affinities will be converted to higher/lower normalized values.

**TABLE 2:** Data statistics.

Variables	Count
Entries	202,510
Alleles	128
Peptides	53,253

## 4 Definitions and Notations

All the key definitions and notations are listed in Table 3.

**TABLE 3:** Notations.

**Notation**	**Meaning**
Peptides and alleles	
*p*	A peptide
*a*	An amino acid of a peptide sequence
P	A set of peptides
Q	An allele
Binding	
*x*/*b*	Original/normalized binding affinity for a peptide-MHC pair
*l*	Binding level for a peptide-MHC pair
Embeddings	
***e***	Encoding vector of amino acid type
***r***	Embedding vector of each amino acid
***o***	Position embedding of each *k*-mer amino acids
*R*	Feature matrix for a peptide sequence
*F*_*G*_	Feature matrix for a padded peptide sequence (i.e., input of global kernel in SpConvM)
Parameters	
*S*(·)	A scoring function
*d*_*e*_	Dimension of amino acid embedding
*d*_*f*_	Number of filters in convolution layer
*d*_*o*_	Dimension of position embedding o
*d*_*g*_	Number of global kernels in SpConvM
*d*_*r*_	Dimension of hidden units c in fully connected layer
*k*	Kernel size in convolutional neural layer
*w*	Attention weight learned in attention layer

## 5 Methods

We developed two new models: ConvM and SpConvM (will be discussed in Sections 5.1 and 5.2), and compare them with MHCflurry (O’Donnell et al., 2018), where MHCflurry is the state-of-the-art and used as the baseline. In terms of the embeddings of amino acids, we compare the performance of SpConvM with three embedding methods for amino acids and their combinations. In terms of the loss functions, we developed three pair-wise hinge loss functions, and compare them with the conventional mean-square loss function used in MHCflurry.

### 5.1 Convolutional Neural Networks with Attention Layers (ConvM)

**FIGURE 1:**
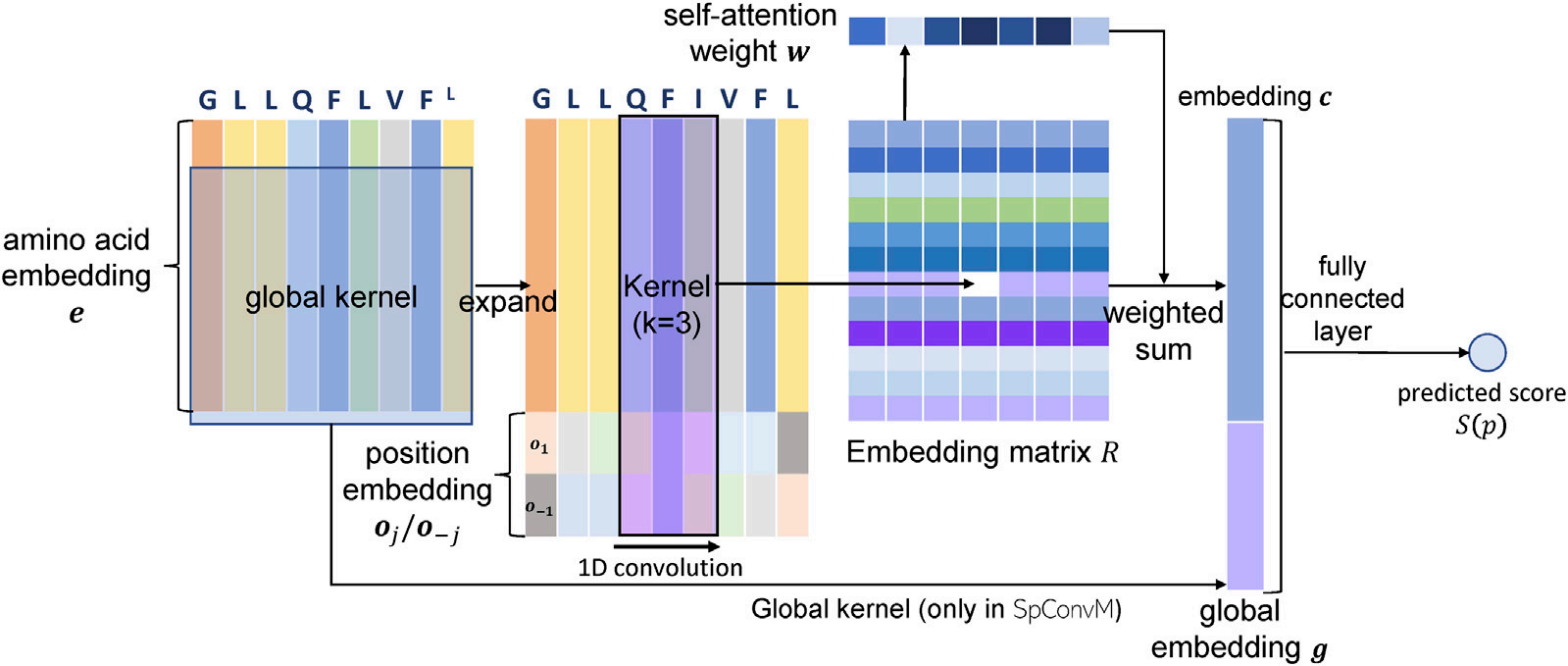
Architectures of ConvM and SpConvM .

In this section, we introduce our new model ConvM, a convolutional neural network with attention layers. Figure 1 presents the architecture of ConvM.

#### 5.1.1 Peptide Representation in ConvM

In ConvM, we first represent each amino acid, denoted as *a*_*j*_, in a peptide sequence, denoted as $p=\left[a_{1},\ldots ,a_{j},\ldots ,a_{n}\right]$ (started from C-end), using two types of information. The first information encodes the type of amino acids using BLOSUM62 matrix or Onehot encoding. The details about the encoding of amino acids are described in Section 6.1.1. The second information encodes the position of each amino acid in peptide sequences, as it has been demonstrated (O’Donnell et al., 2018) that different positions of peptides contribute differently to their binding to alleles. In particular, each position of the peptide sequences, regardless of which amino acid is at the position, will have two position vectors: for the *j*th position from the C-end, we use $o_{j}$ and $o_{-j}\in {\mathbb{R}}^{d_{o}\times 1}$ to represent the position information with respect to the C-end and the N-end, respectively. The two position vectors will together accommodate the variation of peptide lengths. Thus, each amino acid *a*_*j*_ is represented as a feature vector $f_{j}=\left[e_{j};o_{j};o_{-j}\right]\in {\mathbb{R}}^{\left(d_{e}+2d_{o}\right)\times 1}$, where $e_{j}\in {\mathbb{R}}^{d_{e}\times 1}$ is $a{\;}_{j}$’s embedding with an encoding method in Section 6.1.1, and $d_{o}\;$ is the dimension of position embedding vectors; a peptide of *n* amino acids is represented as a feature matrix $F=\left[f_{1},f_{2},\ldots ,f_{n}\right]\in {\mathbb{R}}^{\left(d_{e}+2d_{o}\right)\times n}$. The position vectors will be learned in order to optimize the peptide representations.

#### 5.1.2 Model Architecture of ConvM

The ConvM model consists of a 1D convolutional layer, a self-attention layer and a fully-connected layer as demonstrated in Figure 1. The 1D convolutional layer takes the peptide feature matrix $F\in {\mathbb{R}}^{\left(d_{e}+2d_{o}\right)\times n}$ as input, and extracts local feature patterns from the peptide sequences via 1D convolution using *d*_*r*_ kernels of size $\left(d_{e}\;+2d_{o}\;\right)\times k$. The output of the 1D convolutional layer is an embedding matrix $R=\left[r_{1},\ldots ,r_{\left(n-k+1\right)}\right]\in {\mathbb{R}}^{d_{r}\times \left(n-k+1\right)}$, in which each column *r*_*i*_ represents the embedding of the *i*th *k*-mer out of the *d*_*r*_ kernels. Batch normalization is applied to fix the mean and variance of the embedding matrix *R* in each batch. After batch normalization, rectified linear unit (ReLU) activation is applied as the non-linear activation function on the embedding matrix. Then, we apply the self-attention mechanism (Chorowski et al., 2015) to convert the embedding matrix into an embedding vector ***c*** for the input peptide as follows. First, the weight *w*_*i*_ for *i*th *k*-mer is calculated as follows,

$$w_{i}=\frac{\exp\left(a_{i}\right)}{\sum_{j}\exp\left(a_{j}\right)},a_{i}=v\tanh\left(Wr_{i}+b\right),$$ (2)

where $W\in {\mathbb{R}}^{d_{a}\times d_{r}}$, $b\in {\mathbb{R}}^{d_{a}\times 1}$ and $v\in {\mathbb{R}}^{1\times d_{a}}$ are the parameters of self-attention layer, and $d_{a}\;$ is the number of hidden units in the self-attention layer. With the weight $w_{i}$ on each *k*-mer, the embedding of the whole sequence is calculated as the weighted sum of all *k*-mer embeddings, that is,

$$c=\sum_{i}^{n-k+1}w_{i}r_{i}.$$ (3)

The embedding vector $c\in {\mathbb{R}}^{d_{r}\times 1}$ of the input peptide is then fed into the fully-connected layer to predict peptide binding at the output layer. We will discuss the loss function used at the output layer later in Section 5.3.

### 5.2 Convolutional Neural Networks with Global Kernels and Attention Layers (SpConvM)

We further develop ConvM into a new model with global kernels, denoted as SpConvM as in Figure 1. The use of global kernels is inspired by Bhattacharya et al. (2017), which demonstrates that global kernels within CNN models can significantly improve the performance of peptide binding prediction. As ConvM primarily extracts and utilizes local features, the additional global kernels extract global features from the entire peptide sequences that could be useful for prediction but cannot be captured by local convolution. In order to use global kernels, we pad the peptide sequences of various lengths to length 15, with padding 0 vectors in the middle of the peptide representations in a same way as in MHCflurry. More details about padding are available later in Section 6.1.2. The padded peptide sequences will be encoded into a feature matrix $F_{G}$ in the same way as in ConvM, except that the position embeddings are not included because the global kernels will overwrite the local information after the convolution.

Given the input *F*_*G*_, the convolution using *d*_*g*_ global kernels will generate a vector $g\in {\mathbb{R}}^{d_{g}\times 1}$. We concatenate ***g*** and ***c*** as in ConvM (i.e., the embedding vector calculated from local kernels) to construct a local–global embedding vector ***c***′=[***c***; ***g***] for the input peptide sequence and feed ***c***′ into the fully-connected layer to predict peptide prediction as in Figure 1.

### 5.3 Loss Functions

We propose three pair-wise hinge loss functions, denoted as H_*v*_, H_*l*_ and H_*i*_, respectively. We will compare these loss functions with the widely used mean-square loss function (O’Donnell et al., 2018), denoted as MS, in learning peptide bindings.

#### 5.3.1 Hinge Loss Functions for Peptide Binding Ranking

We first evaluate the hinge loss as the loss function in conjunction with various model architectures. The use of hinge loss is inspired by the following observation. We noticed that in literature, peptide-MHC binding prediction is often formulated into either a regression problem, in which the binding affinities between peptides and alleles are predicted, or a classification problem, in which whether the peptide will bind to the allele is the target to predict. However, in practice, it is also important to first prioritize the most promising peptides with acceptable binding affinities for further assessment, whereas regression and classification are not optimal for prioritization. Besides, recent work has already employed several evaluation metrics on top ranked peptides, for example, (Zeng and Gifford, 2019) evaluated the performance through the true positive rate at 5% false positive rate, which suggests the importance of top-ranked peptides in addition to accurate affinity prediction. All of these inspire us to consider ranking based formulation for peptide prioritization.

Given two normalized binding affinity values b_*i*_ and b_*j*_ of any two peptides *p*_*i*_ and *p*_*j*_ with respect to an allele, the allele-specific pair-wise ranking problem can be considered as to learn a scoring function S(·), such that

$$S\left(p_{i}\right)>S\left(p_{j}\right),\;\;\mathrm{if}\;b_{i}>b_{j}.$$ (4)

Please note that *S*(*p*_*i*_) is a score for peptide *p*_*i*_, which is not necessarily close to the binding affinity *b*_*i*_, as long as it reconstructs the ranking structures among all peptides. This allows the ranking based formulation more flexibility to identify the most promising peptides without accurately estimating their binding affinities. To learn such scoring functions, hinge loss is widely used, and thus we develop three hinge loss functions to emphasize different aspects during peptide ranking.

##### 5.3.1.1 Value-Based Hinge Loss Function

The first hinge loss function, denoted as H_*v*_, aims to well rank peptides with significantly different binding affinities. Given two peptides *p*_*i*_ and *p*_*j*_, this hinge loss function is defined as follows:

$$H_{v}\left(p_{i},p_{j}\right)=\max\left(0,c+\left(b_{i}-b_{j}\right)-\left(S\left(p_{i}\right)-S\left(p_{j}\right)\right)\right),\;\text{where}\;\;l_{i}>l_{j},$$ (5)

where *l*_*i*_ denotes the binding level of peptide *p*_*i*_ according to the Table 1; *l*_*i*_ > *l*_*j*_ denotes that the binding level of peptide *p*_*i*_ is higher than the peptide *p*_*j*_; *b*_*i*_ and *b*_*j*_ are the ground-truth normalized binding affinities of *p*_*i*_ and *p*_*j*_, respectively; *c* > 0 is a pre-specified constant to increase the difference between two predicted scores. *H*_*v*_ learns from two peptides of different binding levels and defines a margin value between two peptides as the difference of their ground-truth binding affinities *b*_*i*_ − *b*_*j*_ plus a constant *c*. If two peptides *p*_*i*_ and *p*_*j*_ are on different binding levels *l*_*i*_ > *l*_*j*_, and the difference of their predicted scores is smaller than the margin *c* + (*b*_*i*_ − *b*_*j*_), this pair of peptides will contribute to the overall loss; otherwise, the loss of this pair will be 0. Note that *H*_*v*_ is only defined on peptides of different binding levels. For the peptides with the same or similar binding affinities, *H*_*v*_ allows incorrect ranking among them.

##### 5.3.1.2 Level-Based Hinge Loss Function

Instead of ranking with respect to the margin as in *H*_*v*_, we relax the ranking criterion and use a margin according to the difference of binding levels (Table 1). Thus, the second hinge loss, denoted as *H*_*l*_, is defined as follows:

$$H_{l}\left(p_{i},p_{j}\right)=\max\left(0,r\times \left(l_{i}-l_{j}\right)-\left(S\left(p_{i}\right)-S\left(p_{j}\right)\right)\right),\;\mathrm{where}\;l_{i}>l_{j},$$ (6)

where *r* > 0 is a constant. Given a pair of peptides in two different binding levels, similar to H_*v*_, H_*l*_ requires that if the difference of their predicted scores is smaller than a margin, this pair of peptides will contribute to the overall loss; otherwise, the loss of these two peptides will be 0. However, unlike H_*v*_, the margin defined in H_*l*_ depends on the difference of binding levels between two peptides (i.e., $r\times \left(l_{i}-l_{j}\right)$). Therefore, in H_*l*_, the margin values of all the peptides $\left(p_{1},p_{2},\ldots ,p_{n}\right)$ on the level *l*_*i*_ to any other peptides on the level *l*_*j*_ will be the same (i.e., $r\times \left(l_{i}-l_{j}\right)$). Note that H_*l*_ is defined on peptides of different binding levels, and thus also allows incorrect ranking among peptides of same binding levels as in ${\text{H}}_{v}$ ; the difference with H_*v*_ is on how the margin is calculated.

##### 5.3.1.3 Constrained Level-Based Hinge Loss Function

The third hinge loss function H_*i*_ extends H_*l*_ by adding a constraint that two peptides of a same binding level can have similar predicted scores. This hinge loss is defined as follows:

$${\text{H}}_{i}\left(p_{i},p_{j}\right)=\{\begin{array}{cc} \text{max}\left(0,r\times \left(l_{i}-l_{j}\right)-\left(S\left(p_{i}\right)-S\left(p_{j}\right)\right)\right), & \text{if }l_{i}>l_{j},\\ \text{max}\left(0,\left|S\left(p_{i}\right)-S\left(p_{j}\right)\right|-r\right), & \text{if }l_{i}=l_{j}. \end{array}$$ (7)

Given a pair of peptides on a same binding level, the added constraint (the case if *l*_*i*_ = *l*_*j*_) requires that if the absolute difference $\left|S\left(p_{i}\right)-S\left(p_{j}\right)\right|$ is smaller than the pre-specified margin *r*, the loss will be zero; otherwise, this pair will have non-zero loss. The constraint on the absolute difference allows incorrect ranking among peptides on a same binding level as long as their predicted scores are similar.

#### 5.3.2 Mean-Squares Loss

We also compare a mean-squares loss function, denoted as MS, proposed in (O’Donnell et al., 2018; Paul et al., 2019), to fit the entries without exact binding affinity values as below:

$$MS\;\left(p{\;}_{i}\right)=\{\begin{array}{cc} {\left(S\left(p_{i}\right)-b_{i}\right)}^{2} & \mathrm{if}\;m_{i}\text{ is quantitative},\\ {\left(\max\left(0,S\left(p_{i}\right)-b_{i}\right)\right)}^{2} & \mathrm{if}\;m_{i}\text{ is qualitative and }\;l_{i}=1\;\;\left(\text{i}\text{.e}\text{., negative binding}\right),\\ {\left(\max\left(0,b_{i}-S\left(p_{i}\right)\right)\right)}^{2} & \mathrm{if}\;m_{i}\text{ is qualitative and }\;l_{i}>1\;\left(\text{i}\text{.e}\text{.},\text{positive binding}\right), \end{array}$$ (8)

where “$m{\;}_{i}\;\text{is quantitative}$” denotes that the peptide-MHC entry *m*_*i*_ is associated with an exact binding affinity value *x*_*i*_. In this case, the MS loss is calculated as the squared difference between the predicted score *S*(*p*_*i*_) and *b*_*i*_ (*b*_*i*_ is normalized from *x*_*i*_ as in Eq. 1). In Eq. 8, “*m*_*i*_ is qualitative” denotes that *m*_*i*_ is associated with a binding level *l*_*i*_ instead of a binding affinity value (Table 1). In this case, *b*_*i*_ is normalized from the binding level thresholds (i.e., $x_{i}\in \left\{100,500,1000,5000\right\}$ in calculating *b*_*i*_ in Eq. 1). When qualitative *m*_*i*_ has *l*_*i*_ = 1, that is, the peptide does not bind to the allele and the binding affinity is low (i.e., large binding affinity value), the predicted score *S*(*p*_*i*_) should be small enough compared to v in order not to increase the loss. When quantitative *m*_*i*_ has $l_{i}>1$, that is, the peptide binds to the allele with reasonably high binding affinity (i.e., small binding affinity value), the predicted score *S*(*p*_*i*_) should be large enough compared to *b*_*i*_ in order not to increase the loss.

Note that in MS, the predicted score *S*(*p*) needs to be normalized into range [0,1]. This is because *b* is in range [0,1] (Eq. 1) so that *S*(*p*) needs to be in the same range and thus neither *S*(*p*) nor *b* will dominate the squared errors due to substantially large or small values. However, in the three hinge loss functions (Eqs. 5–7), the potential different range between *S*(*p*) and *b* or *l* could be accommodated by the constant *c* (Eq. 5) or *r* (Eqs. 6, 7), respectively. In MS, we use sigmoid function to normalize *S*(*p*).

## 6 Experimental Settings

### 6.1 Baseline Methods

#### 6.1.1 Encoding Methods

Encoding methods represent each amino acid with a vector. Popular encoding methods used by the previous works include BLOSUM encoding (Nielsen and Andreatta, 2016; Jurtz et al., 2017; O’Donnell et al., 2018), Onehot encoding (Bhattacharya et al., 2017; Phloyphisut et al., 2019) and Word2Vec embedding method (Vang and Xie, 2017). BLOSUM encoding utilizes the BLOSUM62 matrix (Henikoff and Henikoff, 1992), which measures the evolutionary divergence information among amino acids. We use the *i*th row of the BLOSUM matrix as the feature of *i*th amino acid. Onehot encoding represents the *i*th natural amino acid with an one-hot vector, in which all elements are ‘0’ except the *i*th position as ‘1’. Word2Vec learns the embeddings of amino acids from their contexts in protein sequences or peptide sequences. This embedding method requires learning on a large corpus of amino acid sequences, and is much more complicated than Onehot. However, it is demonstrated (Phloyphisut et al., 2019) that Word2Vec embedding method is comparable to Onehot encoding method, and therefore, we use BLOSUM encoding and Onehot encoding, but not Word2Vec. Besides the above encoding methods, we also evaluate another deep encoding method, denoted as Deep, in which the encoding of each amino acid is learned during the training process. Deep encoding is not deterministic and is learned during the training process; the dimension of embedding vector needs to be specified as a predefined hyper-parameter. We also combine different representations of amino acid generated by the above three encoding methods. These combinations include BLOSUM +Onehot, BLOSUM +Deep, Onehot +Deep and BLOSUM +Onehot +Deep , where “+” represents concatenation of the embeddings of amino acid from different encoding methods.

#### 6.1.2 Baseline Method: Local Connected Neural Networks MHCflurry

MHCflurry (O’Donnell et al., 2018) is a state-of-the-art deep model with locally-connected layers for peptide binding prediction. In MHCflurry, all peptides of length 8 to 15 are padded into length 15 by keeping the first and last four residues and inserting the padding elements in the middle (e.g., ”GGFVPNMLSV” is padded to ”GGFVXXPNXXXMLSV”). The padded sequences are encoded into a feature matrix $E\in {\mathbb{R}}^{15\times 20}$ using BLOSUM encoding method. MHCflurry employs locally-connected layers to extract local feature patterns for each *k*-mers in peptide sequences. Unlike CNN using common filters across all *k*-mer residues in peptides, locally-connected layers apply local filters for each *k*-mer to encode the position-specific features. The encoded feature embeddings for all *k*-mers are then concatenated into a vector, and fed into the fully-connected layer for binding prediction. To the best of our knowledge, MHCflurry is one of the best neural network model for allele-specific peptide binding prediction problem.

Note that we did not compare with other methods including NetMHC4.0, PUFFIN and MHCAttnet on the IEDB dataset. It has been demonstrated in literature (Bonsack et al., 2019) that MHCflurry outperforms or is comparable with NetMHC4.0. We did not compare with NetMHC4.0 because its source code and optimal parameters are not publicly available (they only provided their trained model), and we were not able to reproduce their results using our data. In addition, it is unfair to apply their provided models to test our test set, because it is possible that our test set is included in their training data and thus the performance on our test set can be overestimated. PUFFIN and MHCAttnet are pan methods and take both the peptide and allele sequences as input, but our methods are allele specific.

### 6.2 Batch Generation

For models with MS as the loss function, we randomly sample a batch of peptides as the training batch. For models with the proposed pair-wise hinge loss functions (H_*v*_, H_*l*_, H_*i*_), to reduce computational costs, we construct pairs of peptides for each training batch from a sampled batch of peptides. Specifically, for H_*v*_ and H_*l*_, each pair consists of two peptides from different binding levels; and for H_*i*_, the constructed pairs can consist of two peptides from the same or different binding levels.

### 6.3 Model Training

We use 5-fold cross validation (Bishop, 2006) to tune the hyper-parameters of all methods through a grid search approach. We use 10% of the training data as a validation set and explicitly ensure that the training set, validation set and testing set do not overlap. This validation set is applied to adjust the learning rate dynamically and determine the early stopping of training process. If the loss on the validation set does not decrease in 5 epochs, we will decrease the learning rate by 10%. The learning rate is initialized as 0.05. If the loss does not decrease on the validation set for continuous 20 epochs, we stop the training process. For each allele, we run the grid search algorithm to find the optimal hyper-parameters for the allele-specific model through the above cross validation process. We apply stochastic gradient descent (SGD) to optimize the loss functions. We set the dimension of Deep encoding method as 20, which is equal to the dimension of BLOSUM and Deep encoding method. We also set both the constant *c* in H_*v*_ and the constant *r* in H_*l*_ and H_*i*_ as 0.2.

### 6.4 Evaluation Metrics

We use 4 types of evaluation metrics, including average rank (AR), hit rate (HR), area under the roc curve (AUC), and ROC, to evaluate the performance of the various model architectures and loss functions. Both AR and HR metrics are employed to measure the effectiveness of our model on the prioritization of promising peptides. Specifically, AR metric measures the average overall rankings of promising peptides; HR metric measures the ratio of promising peptides ranked at top; AUC metric measures the possibility that positive peptides are ranked higher than negative peptides; ROC metric measures the ratio of positive peptides that are prioritized higher than top-*n* false positive peptides. We denote $s_{i}$ as the rank of peptide $p_{i}\;$ based on their predicted scores, $P{\;}_{h}$ as the set of peptides with binding affinities smaller than *h* (e.g., *h* = 500 nM). Then *AR*_*h*_ (e.g., *AR*_500_) is defined as follows,

$$AR_{h}=\frac{\sum_{p_{i}\in P_{h}}s_{i}}{\left|P_{h}\right|},\;\text{where }\;P_{h}=\left\{p_{i}|\forall b_{i}<h\right\},$$ (9)

where $\left|P_{h}\right|$ is the size of $P_{h}$. Smaller values of *AR*_*h*_ indicate that promising peptides are ranked higher in general, and thus better model performance.

The hit rate HR_*h*_ (e.g., HR_500_) is defined as follows,

$$HR_{h}=\frac{\left|P_{t}\cap^{\text{​}}P_{h}\right|}{\left|P_{h}\right|},\text{ where }t=\left|P_{h}\right|$$ (10)

where $P_{t}$ denotes the set of peptides with predicted scores ranked at top *t*. Larger values of HR_*h*_ indicate that more promising peptides are prioritized to top-*t* by the model, and thus better performance.

We use *h* = 500 nM as the threshold to distinguish positive peptides and negative peptides, and apply two metrics for classification to evaluate the model performance. The first classification metric AUC is calculated as below,

$$\mathrm{AUC}=\frac{1}{\left|P_{500}\right|\left(\left|P\right|-\left|P_{500}\right|\right)}\sum_{i=1}^{\left|P_{500}\right|}\sum_{j=1}^{\left|P\right|-\left|P_{500}\right|}1\left(S\left(P_{i}\right)>S\left(P_{j}\right)\right),$$ (11)

where P is the set of all peptides, and |P| is the number of peptides in the dataset; $P_{500}$ is the set of all positive peptides, and $\left|P_{500}\right|$ is the number of positive peptides; 1(·) is an indicator function (1(*x*) = 1 if *x* is true, otherwise 0). Larger values of AUC indicate that positive peptides are more likely to be ranked higher than negative peptides. $\mathrm{ROC}{\;}_{t}$ (e.g., $\mathrm{RO}C_{5}$) score is the area under the roc curve up to *t* false positives. $\mathrm{ROC}{\;}_{t}$ is calculated as below.

$${\mathrm{ROC}}_{t}=\frac{1}{\left|P_{500}\right|t}\sum_{i=1}^{\left|P_{500}\right|}\sum_{j=1}^{t}1\left(S\left(P_{i}\right)>S\left(P_{j}\right)\right)$$ (12)

Larger values of $\mathrm{ROC}{\;}_{t\;}$ indicate that the model can prioritize more positive peptides up to first t false positive peptides. We use 7 metrics constructed from the above 4 types of metrics to evaluate the model performance. These 7 metrics include $AR_{100}$, $HR_{100}$, $AR_{500}$, $HR_{500}$, AUC, $\mathrm{RO}C_{5}$ and $\mathrm{RO}C_{10}$.

In order to compare the models with respect to one single metric in a holistic way, we define a hybrid metric H by combining all the evaluation metrics. Given a model trained with a set of hyper-parameters Y, we denote its performance on metric “mtrc ” (mtrc =$\mathrm{AR}{\;}_{h}$, $\mathrm{HR}{\;}_{h}$, $\mathrm{AUC}{\;}_{h}$, $\mathrm{ROC}{\;}_{h}$) as mtrc (Y), and the best metric value as ${\text{best}}_{Y}\left(\text{mtrc}\;\right)={\text{max}}_{Y}\left(\text{mtrc}\;\left(Y\right)\right)$. Then, the hybrid metric H for a model with hyper-parameters Y is defined as below,

$$H\left(Y\right)=\underset{\text{mtrc}}{\sum }\frac{I\left(↓\text{mtrc}\right)\times \left(\text{mtrc}\left(Y\right)-{\text{best}}_{Y}\left(\text{mtrc}\right)\right)}{{\text{best}}_{Y}\left(\text{mtrc}\right)},$$ (13)

where I(↓mtrc) is an identity function: I(↓mtrc)=+1 if smaller values on metric mtrc  indicate better performance; I(↓mtrc)=−1 otherwise. For metrics $AR_{100}$ and $AR_{500}$, a smaller value represents a better model performance.

## 7 Experimental Results

We present the experimental results in this section. All the parameters used in the experiments are reported in the Appendix.

### 7.1 Model Architecture Comparison

We evaluate all the 12 possible combinations of the 3 model architectures (ConvM, SpConvM, MHCflurry) and the 4 loss functions (${\text{H}}_{v}$, ${\text{H}}_{l}$, ${\text{H}}_{i}$, MS) with all the encoding methods through 5-fold cross validation. Table 4 presents the overall performance comparison with BLOSUM +Onehot +Deep  encoding method (encoding method comparison will be presented later in Section 7.3). We apply the grid search to determine the optimal hyperparameters of each method on each allele with respect to the hybrid metric *H* (Appendix Section A1.1), and report the best performance in Table 4. We use MHCflurry with MS loss in (O’Donnell et al., 2018) as the baseline, and calculate the percentage improvement of our methods over the baseline across 128 alleles. In Table 4, the best model for each allele is selected with respect to *H*; the model performance is further evaluated using the 7 evaluation metrics.

**TABLE 4:** Overall performance comparison (*H*; BLOSUM +Onehot +Deep ).

**Model**	**Loss**	$\mathrm{AR}{\;}_{100}$	$\mathrm{HR}{\;}_{100}$	$\mathrm{AR}{\;}_{500}$	$\mathrm{HR}{\;}_{500}$	AUC	$\mathrm{ROC}{\;}_{5}$	$\mathrm{ROC}{\;}_{10}$
ConvM	${\text{H}}_{v}$	7.93	4.71	2.80	5.48	5.13	8.43	7.26
	${\text{H}}_{l}$	5.63	5.47	1.66	3.59	4.56	7.11	4.65
	${\text{H}}_{i}$	6.35	5.70	0.99	2.59	4.16	4.69	4.42
	MS	−6.26	0.02	−7.87	−3.98	0.16	−3.34	−3.94
SpConvM	${\text{H}}_{v}$	**11.58**	**10.47**	**7.28**	**8.28**	**6.70**	**17.10**	**14.42**
	${\text{H}}_{l}$	8.97	8.64	6.57	7.36	6.04	12.89	10.85
	${\text{H}}_{i}$	10.01	8.87	4.73	6.00	6.00	14.01	11.36
	MS	8.66	8.14	2.77	4.28	3.93	13.54	9.68
MHCflurry	${\text{H}}_{v}$	11.06	8.93	5.60	5.20	4.42	11.10	9.51
	${\text{H}}_{l}$	9.45	5.77	5.09	4.43	4.72	8.05	6.95
	${\text{H}}_{i}$	8.83	6.35	4.54	5.73	4.52	7.10	5.88
	MS	0.00	0.00	0.00	0.00	0.00	0.00	0.00

The values in the table are percentage improvement compared with the baseline MHCflurry with MS. Models are trained using BLOSUM +Onehot +Deep  encoding methods, and selected with respect to H and evaluated using the 7 evaluation metrics. The best improvement with respect to each metric is **bold**.

Table 4 shows that as for the model architectures, on average, SpConvM achieves the best performance overall among all three model architectures (e.g., SpConvM with MS has 8.66% improvement in AR_100_ and 8.14% in HR_100_ over MHCflurry with MS). Please note that when we calculate the improvement, we exclude alleles on which our models achieve more than 150% improvement (typically no more than 15 such alleles under different metrics). This is to remove potential bias due to a few alleles on which the improvement is extremely substantial. SpConvM performs better than ConvM on average. SpConvM extends ConvM with global kernels to extract global features from the entire peptide sequences. The better performance of SpConvM than that of ConvM indicates that global features could capture useful information from entire peptide sequences, which are typically short, for binding prediction. In addition, MHCflurry outperforms ConvM on average. The difference between MHCflurry and ConvM is that MHCflurry learns position-specific features via position-specific kernels, and ConvM learns local features via kernels that are common to all the locations. As demonstrated in other studies (O’Donnell et al., 2018) that certain positions of peptides are more critical for their binding to alleles, the better performance of MHCflurry over ConvM could be attributed to its position-specific feature learning capability. Moreover, since the peptide sequences are usually short (8–15 amino-acid long), it is very likely that these short sequences do not have strong local patterns, and thus ConvM could not capture a lot of useful local information. In comparison with MHCflurry, SpConvM integrates both local features via its ConvM component and global features via global kernels. Such integration could enable SpConvM to capture global information as compensation to local features, and thus to improve model performance.

We also report the results of our methods on 34 HLA-A molecules and 35 HLA-B molecules with the optimal hyperparameters determined by hybrid metric *H* separately in Table 5. HLA-A and HLA-B are two groups of the human leukocyte antigen (HLA) complex that are important to the immune system. The results of HLA-A and HLA-B molecules show the same trend as that in Table 4, that is, for both HLA-A and HLA-B groups, on average, SpConvM still outperforms ConvM and MHCflurry on most metrics (e.g., SpConvM with *H*_*v*_ has 5.71% improvement on $\mathrm{AR}{\;}_{100}$ and SpConvM with MS has 3.02% improvement on $\mathrm{HR}{\;}_{100}$).

**TABLE 5:** Overall performance comparison across HLA-A and HLA-B alleles (H; BLOSUM +Onehot +Deep ).

**Allele**	**Model**	**Loss**	$\mathrm{AR}{\;}_{100}$	$\mathrm{HR}{\;}_{100}$	$\mathrm{AR}{\;}_{500}$	$\mathrm{HR}{\;}_{500}$	AUC	$\mathrm{ROC}{\;}_{5}$	$\mathrm{ROC}{\;}_{10}$
HLA-A	ConvM	${\text{H}}_{v}$	0.56	3.38	1.32	4.68	2.04	2.04	−0.43
		${\text{H}}_{l}$	−3.12	1.06	−2.44	1.12	0.95	−1.27	−3.03
		${\text{H}}_{i}$	−4.23	3.38	−3.41	−2.02	0.76	−3.93	−4.62
		MS	−4.79	1.36	−5.41	−0.22	−0.04	1.71	−0.36
	SpConvM	${\text{H}}_{v}$	**5.71**	**8.35**	**4.01**	1.94	2.61	**7.14**	4.34
		${\text{H}}_{l}$	3.73	2.66	3.10	4.81	**2.83**	6.10	1.79
		${\text{H}}_{i}$	3.28	4.47	3.02	1.90	2.54	4.14	0.51
		MS	−1.40	3.02	−2.74	−0.51	0.84	8.53	3.76
	MHCflurry	${\text{H}}_{v}$	2.75	2.22	2.87	**5.26**	1.87	3.61	**5.22**
		${\text{H}}_{l}$	2.43	1.65	2.39	4.37	2.29	1.30	0.50
		${\text{H}}_{i}$	2.51	1.64	2.21	0.57	1.94	0.11	−0.57
		MS	0.00	0.00	0.00	0.00	0.00	0.00	0.00
HLA-B	ConvM	${\text{H}}_{v}$	**5.12**	**6.98**	0.71	5.38	2.45	11.20	3.87
		${\text{H}}_{l}$	−0.81	−1.63	−1.43	1.57	2.18	5.47	0.19
		${\text{H}}_{i}$	0.27	−2.90	−4.44	3.44	2.29	−0.42	−1.21
		MS	−8.87	2.99	−13.18	−4.75	−1.69	0.75	−4.06
	SpConvM	${\text{H}}_{v}$	4.63	4.45	**6.55**	7.43	3.24	**17.12**	**9.85**
		${\text{H}}_{l}$	3.53	2.37	5.85	**9.20**	3.06	15.48	8.95
		${\text{H}}_{i}$	7.08	−1.05	4.79	9.04	**3.29**	15.77	8.31
		MS	−1.44	−2.19	−3.57	2.56	0.42	8.73	4.16
	MHCflurry	${\text{H}}_{v}$	4.10	5.21	5.04	8.04	2.96	12.14	6.58
		${\text{H}}_{l}$	−2.16	−0.99	3.89	7.41	2.17	11.69	5.31
		${\text{H}}_{i}$	3.51	2.16	3.02	4.78	1.49	7.52	3.05
		MS	0.00	0.00	0.00	0.00	0.00	0.00	0.00

The values in the table are percentage improvement compared with the baseline MHCflurry with MS. Models are trained using BLOSUM +Onehot +Deep  encoding methods, and selected with respect to H and evaluated using the 7 evaluation metrics. The best improvement with respect to each metric is **bold**.

In addition to using the hybrid metric H to determine the optimal hyperparameters, we also apply another four metrics $\mathrm{AR}{\;}_{100}$, $HR_{100}$, AUC and $\mathrm{ROC}{\;}_{5}$ to select the hyperparameters. The results of the best models in terms of these four metrics are presented in Supplementary Appendix Tables A1–A4, respectively. The results show the same trend as that in Table 4, that is, on average, SpConvM outperforms ConvM and MHCflurry on all 7 metrics and *H*_*v*_ loss function is the best among all loss functions.

**FIGURE 2:**
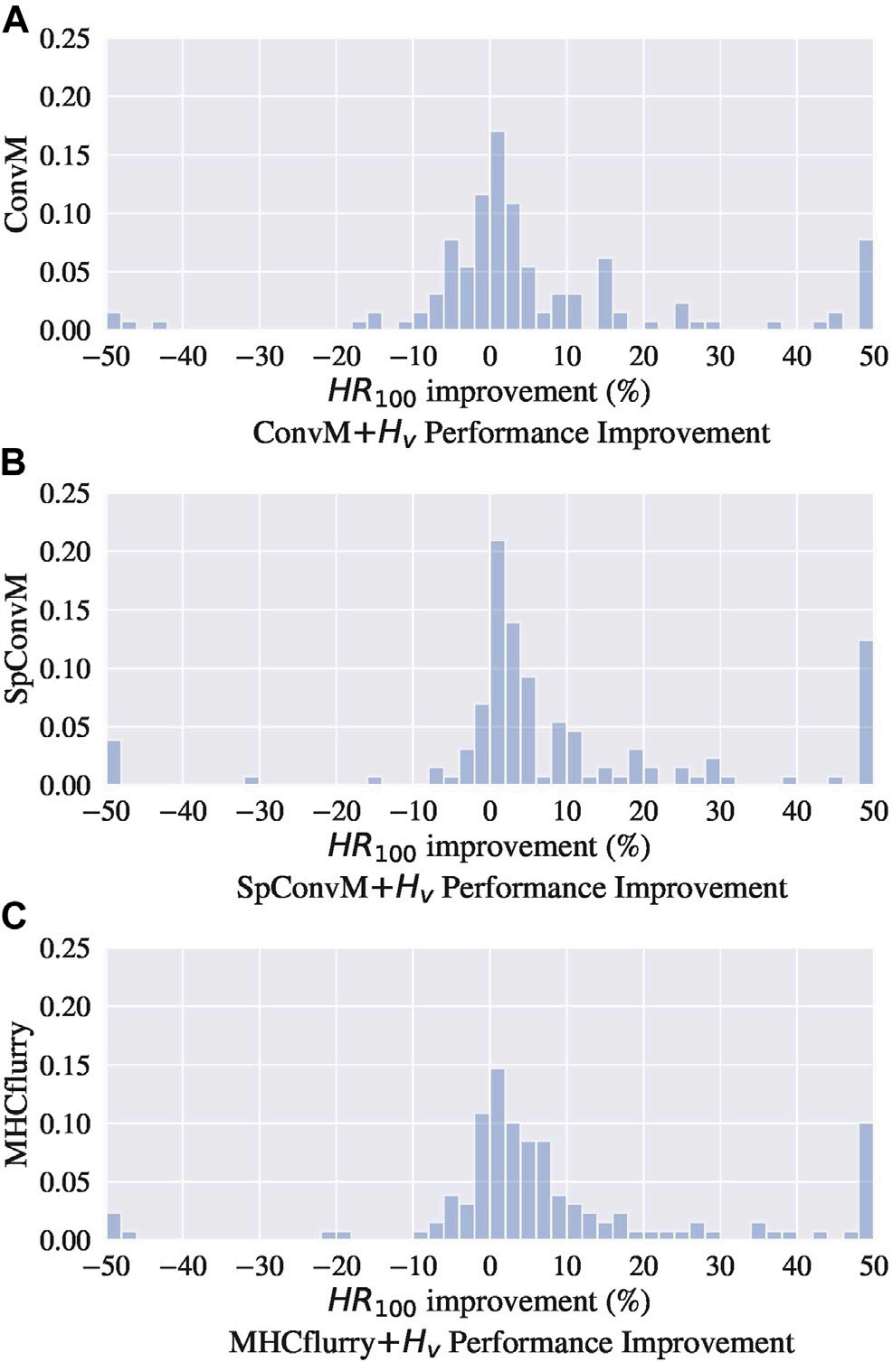
Performance improvement compared with MHCflurry with MS among all Alleles.

Figure 2 show the distributions of performance improvement among all the alleles from ConvM, SpConvM and MHCflurry with *H*_*v*_, in comparison with MHCflurry with MS, respectively. All the methods use BLOSUM +Onehot +Deep  as encoding methods, and the performance is evaluated using $\mathrm{HR}{\;}_{100}$ in a same way as that in Table 4. Figure 2A shows that in ConvM, about half of the alleles have performance improvement compared to that in MHCflurry with MS. Overall, there is an average 4.71% improvement among all the alleles. Figure 2B shows that more alleles have performance improvement in SpConvM compared to that in ConvM and in MHCflurry with MS, and more alleles have significant improvement. This indicates the strong performance of SpConvM. Figure 2C shows that in comparison with MS as the loss function, MHCflurry has more improvement using *H*_*v*_ as the loss function (average improvement 8.93%).

### 7.2 Loss Function Comparison

Table 4 also demonstrates that *H*_*v*_ is the most effective loss function in combination with each of the learning architectures, and all three hinge loss functions *H*_*v*_, *H*_*l*_ and *H*_*i*_ can outperform the MS loss function. For example, for SpConvM, the performance improvement of *H*_*v*_, *H*_*l*_, *H*_*i*_ and MS in terms of $AR{\;}_{100}$ follows the order $H_{v}\left(11.58\%\right)>H_{i}\left(10.01\%\right)>H_{l}\left(8.97\%\right)>MS\left(8.66\%\right)$, compared with the baseline MHCflurry with MS. This trend is also consistent for ConvM and MHCflurry. The results of HLA-A and HLA-B molecules in Table 5 also show the same trend as that in Table 4. The better performance of *H*_*v*_ may be due to the use of a margin in the loss function that is determined by the binding affinity values (Eq. 5). This value-based margin could enforce granular ranking among peptides even when they are from a same binding level. In *H*_*l*_ (Eq. 6) and ${\text{H}}_{i}$ (Eq. 7), the margins are determined based on the levels of the binding affinities. While *H*_*l*_ and *H*_*i*_ can still produce ranking structures of peptides according to their binding levels, they may fall short to differentiate peptides of a same binding level.

All the three hinge loss functions *H*_*v*_, *H*_*l*_ and *H*_*i*_ outperform MS across all the model architectures. This might be due to two reasons. First, the pairwise hinge loss functions are less sensitive to the imbalance of different amounts of peptides, either strongly binding or weakly/non-binding, by sampling and constructing pairs from respective peptides. Thus, the learning is not biased by one type of peptides, and the models can better learn the difference among different types of peptides, and accordingly produce better ranking orders of peptides. Second, the pairwise hinge loss functions can tolerate insignificant measurement errors to some extent. All the three hinge loss functions do not consider pairs of peptides with similar binding affinities. This enables our models to be more robust and tolerant to noisy data due to the measurement inaccuracy of binding affinities.

### 7.3 Encoding Method Comparison

We evaluate three encoding methods (BLOSUM, Onehot, Deep) and their combinations (BLOSUM +Deep, Onehot+Deep, BLOSUM +Onehot, BLOSUM +Onehot+Deep) over SpConvM with ${\text{H}}_{v}$ loss (the best loss function overall) using BLOSUM  through 5-fold cross validation. We report the results of best models across all 128 alleles in the same way as in Section 7.1 (i.e., model selection with respect to H, evaluated using the 7 metrics). Table 6 presents the average percentage improvement of the 7 encoding methods over the baseline on the 7 metrics. The reported results in Table 6 are from the models with the optimal hyperparameters that are selected according to the hybrid metric H. Table 6 shows that BLOSUM +Onehot+Deep encoding method achieves the best performance in general. BLOSUM +Onehot+Deep encodes the amino acids using their inherent evolutionary information via BLOSUM  and identity of different amino acids via Onehot, both of which are deterministic and not specific to the learning problem, and also the allele-specific information via Deep, which is learned in the model and thus specific to the learning problem. The combination of deterministic, amino acid identities and learned features enables very rich information content in the embeddings, and could be the reason why it outperforms others. With a similar rationale, BLOSUM +Deep achieves the second best performance in general. BLOSUM  on its own outperforms Onehot and Deep, respectively, indicating BLOSUM  is rich in representing amino acid information. Combing BLOSUM  with Onehot and Deep, respectively, introduces notable improvement over BLOSUM  alone, indicating that BLOSUM +Onehot and BLOSUM +Deep are able to represent complementary information rather than that in BLOSUM . Onehot on itself alone performs the worst primarily due to its very limited information content. Combing Onehot with Deep improves from Onehot but does not perform well compared to Deep alone. This may be due to that Onehot (i.e., amino acid identity) information still plays a substantial role in Onehot+Deep so Deep information does not supply sufficient additional information.

**TABLE 6:** Encoding performance comparison on SpConvM with ${\text{H}}_{v}$ using BLOSUM  (H).

**Encoding**	$\mathrm{AR}{\;}_{100}$	$\mathrm{HR}{\;}_{100}$	$\mathrm{AR}{\;}_{500}$	$\mathrm{HR}{\;}_{500}$	AUC	$\mathrm{ROC}{\;}_{5}$	$\mathrm{ROC}{\;}_{10}$
BLOSUM	0.00	0.00	0.00	0.00	0.00	0.00	0.00
Onehot	−6.92	−4.38	−4.97	−2.37	−0.73	−5.91	−4.63
Deep	−3.33	0.69	−2.76	−0.56	−0.15	−1.22	−1.63
BLOSUM +Onehot	−0.96	0.95	0.21	0.79	0.3	1.57	0.89
BLOSUM +Deep	**1.37**	**1.79**	0.69	1.49	0.61	3.39	2.49
Onehot+Deep	−4.72	−1.65	−3.37	−1.26	−0.32	−3.25	−2.67
BLOSUM +Onehot+Deep	−0.36	0.11	**1.17**	**2.12**	**0.69**	**4.58**	**3.46**

The values in the table are percentage improvement compared with SpConvM with ${\text{H}}_{v}$ using BLOSUM  . Models are selected with respect to H and evaluated using the 7 evaluation metrics. The best improvement with respect to each metric is **bold**.

We also select the optimal set of hyperparameters with respect to $\mathrm{AR}{\;}_{100}$, $\mathrm{HR}{\;}_{100}$, AUC and $\mathrm{ROC}{\;}_{5}$, and report the corresponding results in Supplementary Appendix Tables A5–A8, respectively. With different model selection metrics, the encoding methods have different performance. However, in general, BLOSUM +Onehot+Deep achieves better performance than other encoding methods over all the metrics.

### 7.4 Attention Weights

**FIGURE 3:**
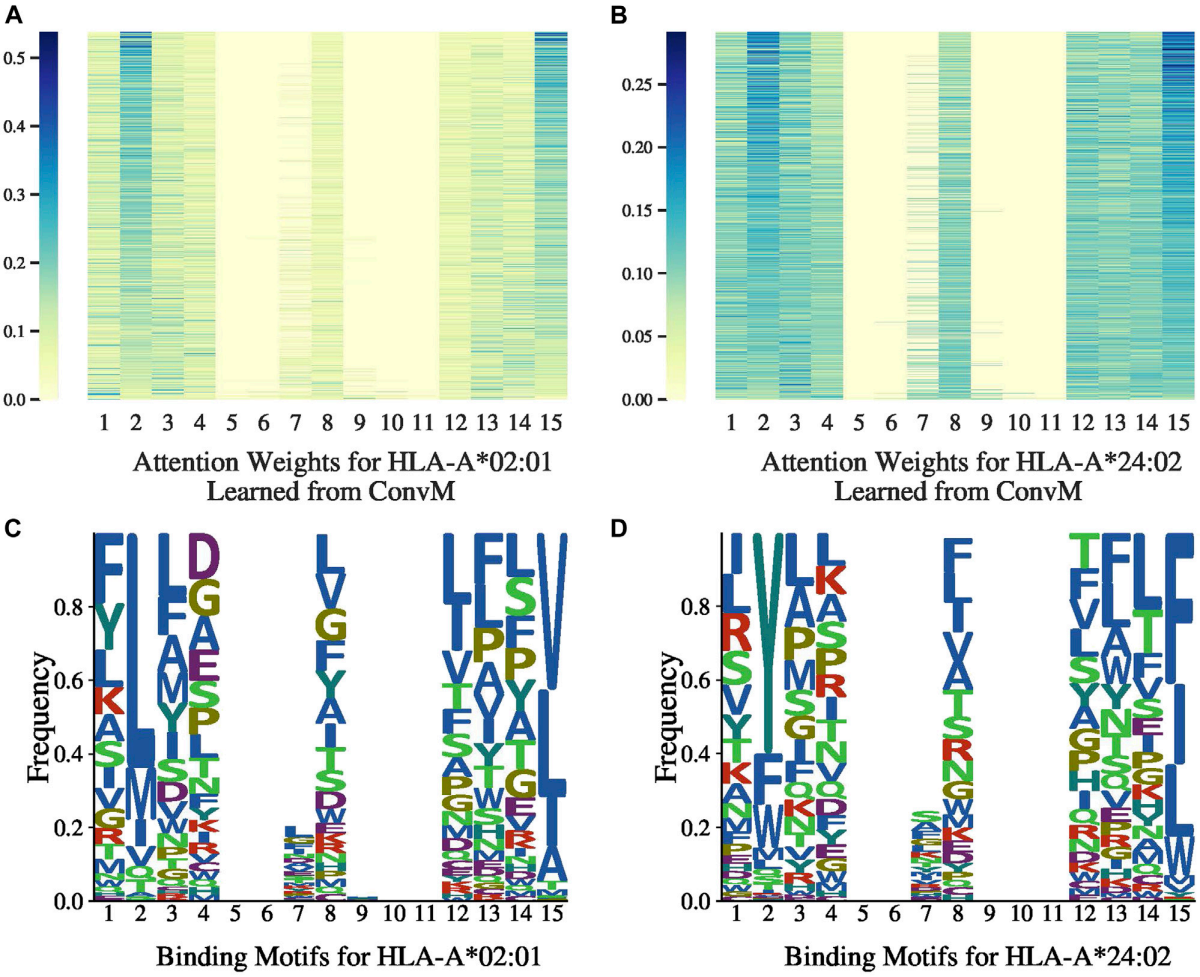
Attention weights and motifs for HLA-A*02:01 and HLA-A*24:02.

**FIGURE 4:**
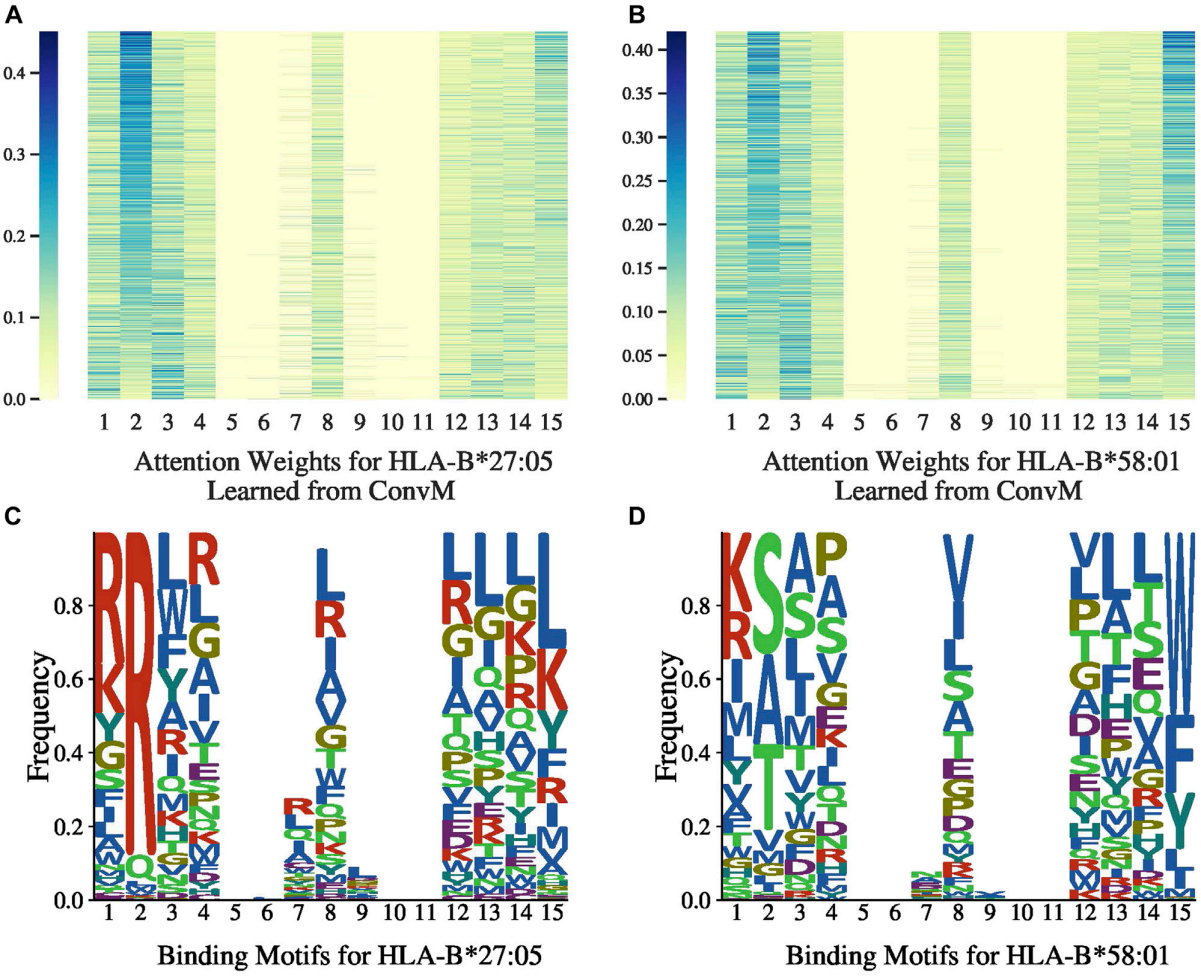
Attention weights and motifs for HLA-B*27:05 and HLA-B*58:01.

Figures 3A,B, 4A,B present the attention weights over peptides of allele HLA-A*02:01, HLA-A*24:02, HLA-B*27:05 and HLA-B*58:01, respectively, learned by the attention layer of ConvM (with BLOSUM +Onehot+Deep, *H*_*v*_; position 5, 6, 7 and 9, 10, 11 are padding positions if the sequence length is less than 15). In these figures, each column represents the weight of 1-mer embedding, that is, the embedding over one amino acid, because the best kernel size for these four alleles in ConvM is 1; each row represents an attention weight learned for a specific peptide by ConvM. Figure 3A shows that for HLA-A*02:01, the amino acids located at the second position and the last position contribute most to the binding events. This is consistent with the conserved motif calculated by SMM matrix (Peters and Sette, 2005). Figures 3A, 4A, and 4B also show the clear position-specific binding patterns for the other three alleles: for HLA-A*24:02, the second and last positions have higher weights; for HLA-B*27:05, the second position has higher weights; and for HLA-B*58:01, the second and last positions have higher weights. This indicates that ConvM with the attention layer is able to accurately learn the importance of different positions in peptides in predicting peptide activities.

Figures 3C,D, 4C,D present the allele-specific binding motifs of the peptides with high affinity for allele HLA-A*02:01, HLA-A*24:02, HLA-B*27:05 and HLA-B*58:01, respectively. Comparing with Figures 3A,B, 4A,B respectively, we noticed that for sequence positions that have higher weights, there are a few preferred amino acids; for positions with lower weights, the amino acids can be diverse. We further check whether the learned attention weights correlate with amino acid conservation. We first calculate a matrix, denoted as $\mathrm{SM}M_{l}$, where each row represents an amino acid, each column represents a position in peptides, and each value in the matrix is calculated as follows,

$$\mathrm{SM}M_{l}\left(a,j\right)=\underset{p\in P}{\sum }I\left(\text{amino acid }a\;\text{ is at }j-\text{th position in }p\;\right)\times w_{j},$$ (14)

where I(x) is the indicator function (I(x)=1 if *x* is true, otherwise 0), and *w*_*j*_ is the learned attention weight at the *j*th position. That is, SMM_*l*_(*a*, *j*) is the sum of attention weights at the *j*th position aggregated from all peptides if amino acid *a* appears at that position in those peptides. We calculated the correlation between the Stabilized Metrix Method (SMM) scoring matrix provided by IDEB (Vita et al., 2018) and SMM_*l*_ for HLA-A*02:01 after they are flattened. The correlation is −0.7613. Since in SMM, smaller values indicate that amino acids are preferred, and in SMM_*l*_, larger values indicate high frequencies (i.e., preferred) with high weights, the negative correction value indicates strong correlation between SMM and SMM_*l*_. Similarly, for HLA-A*24:02, the correlation is −0.6615 and thus also strong. We also calculated the correlation at anchor positions between the SMM and SMM_*l*_ for HLA-A*02:01 and HLA-A*24:02. The correlations for HLA-A*02:01 are −0.8809 at position 2 and −0.8945 at position 9. The correlations for HLA-A*24:02 are −0.9128 at position 2 and −0.6636 at position 9. These strong correlations demonstrate that our learned attention weights are able to indicate motifs represented by SMM.

**FIGURE 5:**
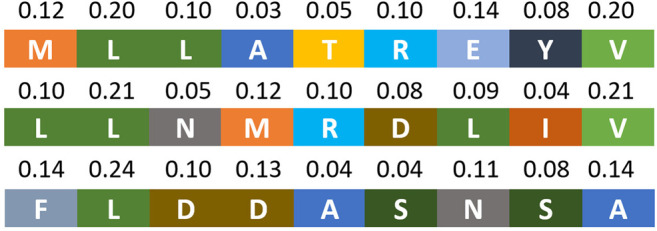
Attention weights of three peptides for HLA-A*02:01 learned from ConvM.

Figure 5 presents the attention weights learned from ConvM of three peptides binding to HLA-A*02:01. In this figure, each line represents a peptide sequence in which each colored block represents an amino acid. The number above each block represents the learned attention weight at that position. Figure 5 shows that our model can capture the anchor position and amino acids that are important to the binding events: the second amino acids and the last amino acids are associated with the largest attention weights.

We do not present the attention weights learned by SpConvM, as in SpConvM, the attention weights do not show binding patterns as clear as those in ConvM. This is due to that SpConvM incorporates both local features and global features, and the global features might significantly contribute to the prediction and therefore the contribution from local features is reduced.

### 7.5 Position Embeddings

**FIGURE 6:**
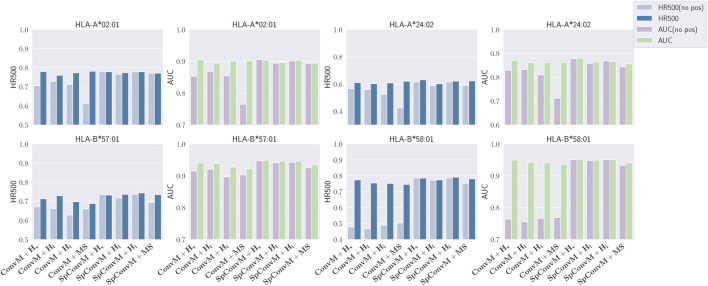
Performance comparison on ConvM and SpConvM with and without position encoding.

We conduct an ablation study to verify the effect of position embedding in the ConvM and SpConvM. We compare the performance of ConvM and SpConvM with and without position embedding on four alleles including: HLA-A*02:01, HLA-A*24:02, HLA-B*57:01 and HLA-B*58:01. We run the combinations of ConvM and SpConvM with 4 loss functions (i.e., *H*_*v*_, *H*_*l*_, *H*_*i*_, MS) using 5-fold cross validation. Figure 6 presents the performance comparison over $\mathrm{HR}{\;}_{500}$ and AUC. In this figure $\mathrm{HR}{\;}_{500}$(no pos) and AUC (no pos) represent model performance in $\mathrm{HR}{\;}_{500}$ and AUC without position embedding, and $\mathrm{HR}{\;}_{500}$ and AUC represent that with position embedding. As demonstrated in Figure 6, position embedding in ConvM can lead to significant performance improvement in terms of $\mathrm{HR}{\;}_{500}$ and AUC. The better performance of ConvM with position embedding also demonstrates the importance of position information to the binding events of peptide-MHC pairs. The performance improvement induced by the position embedding on SpConvM is not as significant as that of ConvM. This is due to that the global kernel in SpConvM can reduce the effect of position embedding, since the global kernel can also encode the absolute position information of each amino acid as position embedding does.

Literature Chorowski et al. (2015) shows that a main limitation of weighted-sum operation in attention layer is that the same *k*-mers with position-unaware embeddings will be associated with the exactly same attention weights regardless of their position in the peptide sequence. Such position-unaware weights are against our knowledge, that is, amino acids in specific positions have been known to be important for binding events O’Donnell et al. (2018); O’Donnell et al. (2020). Hence, encoding position information into the embeddings of *k*-mers is necessary for the self-attention layer to learn meaningful position-specific binding patterns.

### 7.6 Training and Inference Time

We implemented the models using Python-3.6.9, Pytorch-1.3.1 and Numpy-1.18.1. We trained the models on machines with Intel Xeon E5-2680 v4 CPUs and NVIDIA Tesla P100 (Pascal) GPU 16 GB memory with Red Hat Enterprise 7.7. With different hyperparameters, the average training time for each allele with 5-fold cross validation (i.e., 5 models for each allele) is 3.81 min [(3.41, 4.28), standard deviation (±0.34)] with a single CPU core and a single GPU. We also calculated the average inference time for three alleles HLA-A-2402, HLA-A-0201, HLA-A-0301. On average, the inference with our ConvM model takes 0.88 μs per peptide, and the inference with our SpConvM model takes 1.46 μs per peptide.

## 8 Discussions

### 8.1 Experiments on Mass Spectrometry Benchmark Dataset

We evaluated the performance of our methods with the Mass Spectrometry benchmark dataset curated by O’Donnell et al. (2018). This MS benchmark dataset contains 23,653 sequences of MHC-displayed ligands eluted from B cell lines expressing 15 MHC class I alleles. For each eluted ligand, 100 decoys will be sampled from the protein-coding transcripts that contained this eluted ligand. Specifically, they sampled an equal number of decoys of each length 8–15. After removing all the entries present in the IEDB dataset, the yielded Mass Spectrometry benchmark dataset contains 23,653 positive peptides and 2,377,037 randomly sampled negative peptides.

To compare with other methods on the Mass Spectrometry benchmark dataset, we follow the idea of model ensemble that MHCflurry applied on this dataset. Similarly as in MHCflurry, for each of our methods (ConvM and SpConvM with the three hinge loss functions *H*_*v*_, *H*_*l*_ and *H*_*i*_), we train models using 90% samples of IEDB dataset, in which 10% of the training data are randomly sampled as a validation set for early stopping. The remaining 10% data in the IEDB dataset is used as the test set for parameter turning. Note that during model training, we use the same negative sampling method as in MHCflurry to generate negative training peptides, that is, we generate 25 random negative peptides for each of length 8–15 at each epoch. For each set of hyperparamenters, we train 8 models as above with different randomized validation sets but a same test set. The top-16 best performing models are selected to predict on the Mass Spectrometry benchmark dataset. For each allele in the benchmark dataset, the final ranking position of each peptide for that allele is calculated as the geometric mean of its 16 ranking positions out of the 16 best models. Tables 7 and 8 present the performance comparison between our ensemble method, denoted as ConvM−e, and three other state-of-the-art methods on the benchmark data including MHCflurry, NetMHC4.0 and NetMHCpan3.0.

**TABLE 7:** Performance comparison over mass spectrometry dataset in PPV .

**Allele**	MHCflurry	NetMHC4.0	NetMHCpan3.0	ConvM−e
HLA-A*01:01	**0.8055**	0.6578	0.7700	0.7910
HLA-A*02:01	0.7014	0.6182	0.6516	**0.7112**
HLA-A*02:03	**0.7443**	0.7060	0.6984	0.7180
HLA-A*02:07	**0.5566**	0.2645	0.5283	0.4608
HLA-A*03:01	**0.6288**	0.5238	0.5876	0.6267
HLA-A*24:02	**0.7625**	0.6432	0.7257	0.7620
HLA-A*29:02	**0.7355**	0.6334	0.7007	0.7181
HLA-A*31:01	0.4491	0.3989	**0.4649**	0.4209
HLA-A*68:02	**0.5181**	0.4975	0.5096	0.4960
HLA-B*35:01	0.6443	0.6119	**0.6510**	0.6488
HLA-B*44:02	0.7213	0.6952	**0.7623**	0.7577
HLA-B*44:03	**0.7840**	0.6414	0.7478	0.7621
HLA-B*51:01	0.7104	0.6305	0.6248	**0.7368**
HLA-B*54:01	0.6371	0.5882	0.6230	**0.6603**
HLA-B*57:01	0.6223	0.5331	0.5952	**0.6542**

The best performance for each allele is **bold**. The second best performance for each allele is underlined.

**TABLE 8:** Performance comparison over mass spectrometry dataset in AUC.

**Allele**	MHCflurry	NetMHC4.0	NetMHCpan3.0	ConvM−e
HLA-A*01:01	0.9873	0.9854	**0.9881**	0.9864
HLA-A*02:01	**0.9836**	0.9775	0.9798	0.9820
HLA-A*02:03	**0.9903**	0.9888	0.9879	0.9878
HLA-A*02:07	**0.9630**	0.9176	0.9600	0.9339
HLA-A*03:01	**0.9714**	0.9632	0.9648	0.9678
HLA-A*24:02	0.9905	0.9857	0.9895	**0.9917**
HLA-A*29:02	0.9580	0.9549	**0.9651**	0.9648
HLA-A*31:01	0.9276	0.9408	**0.9483**	0.9204
HLA-A*68:02	0.8101	0.8039	**0.8232**	0.7930
HLA-B*35:01	0.8765	0.8786	0.8744	**0.8859**
HLA-B*44:02	0.9796	0.9770	0.9791	**0.9808**
HLA-B*44:03	0.9765	0.9696	0.9723	**0.9768**
HLA-B*51:01	0.9314	0.9275	0.9195	**0.9339**
HLA-B*54:01	0.9255	0.9301	0.9341	0.9264
HLA-B*57:01	0.8799	0.8667	0.8756	**0.8842**

The best performance for each allele is **bold**. The second best performance for each allele is underlined.

Table 7 shows that in terms of PPV (positive predictive value, a popular metric using on the Mass Spectrometry dataset), our ensemble methods achieve either the best or the second best performance on 12 out of 15 alleles among all the methods. When our ensemble achieves the second best performance on an allele, it is very comparable to the best performance—on average, the difference is 0.0112. For HLA-B alleles, our ensemble methods are also the best or the second best. Table 8 shows a similar trend in terms of AUC, that is, our ensemble methods achieve either the best or the second best performance among out 9 of 15 alleles among all the methods; when it is the second best method, its performance is very comparable to the best method. In particular, for HLA-B alleles, our ensemble methods achieve the best performance on 5 out of 6 alleles. The results in the above two tables demonstrate that our methods either outperform the other methods, or are very comparable to the other methods.

#### 8.1.1 Discussion on Using an Independent Test Set

**FIGURE 7:**
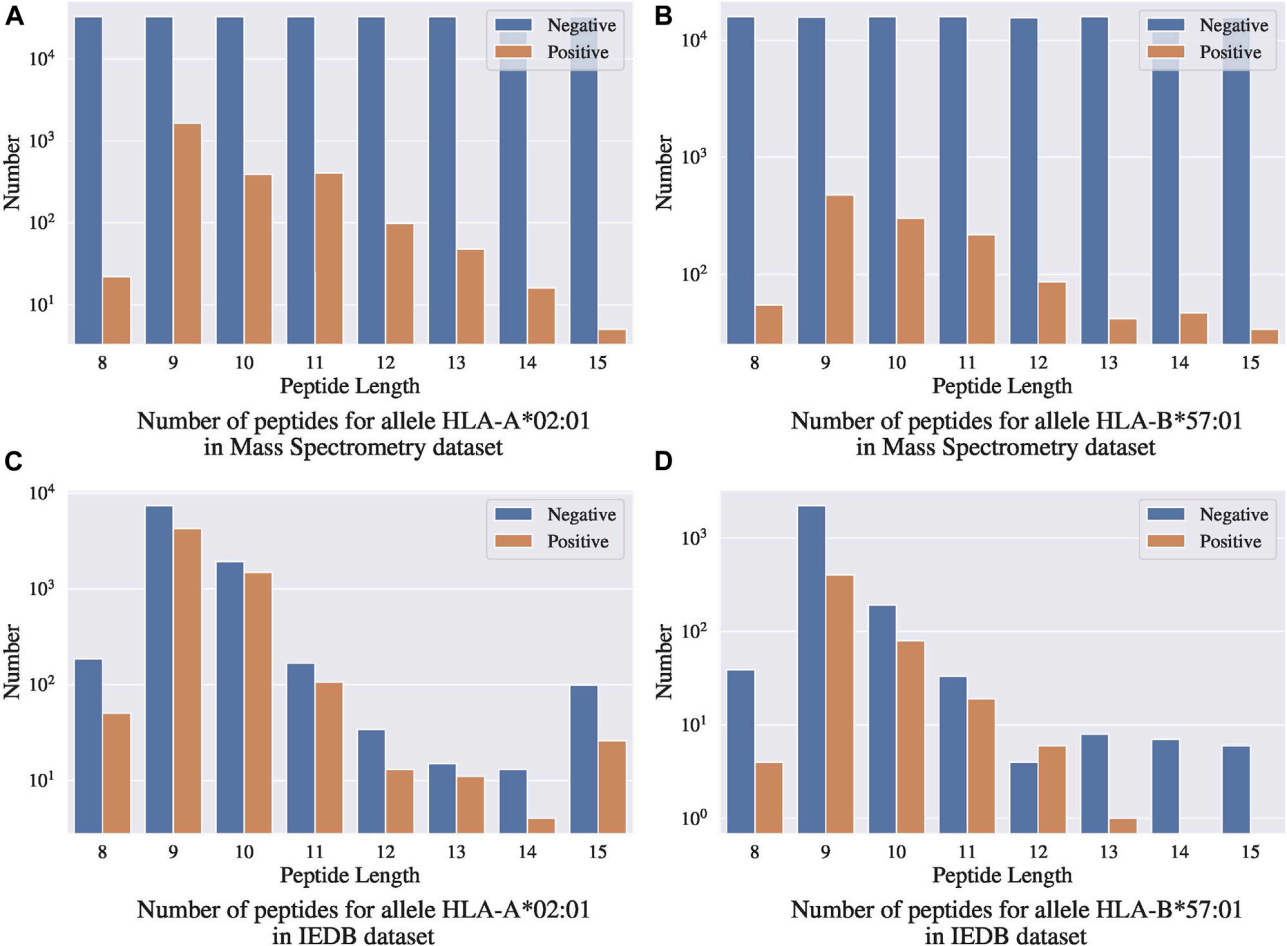
Comparison between mass spectrometry dataset and IEDB dataset.

One concern with this benchmark dataset is that the random negative sampling method creates a data distribution that is different from that of real data. Figures 7A,B present the distributions of binding ligands and the randomly sampled negative samples for two different alleles in the Mass Spectrometry benchmark dataset, respectively; Figures 7C,D present the corresponding distributions in the IEDB dataset. Both the benchmark dataset and the IEDB dataset have similar distributions on the binding peptides, that is, most of the binding peptides have sequence length 9 to 11. In the IEDB dataset, the negative peptides have a similar distribution over sequence lengths as the positive peptides, and the dataset is balanced in terms of positive and negative sample size. However, in the benchmark dataset, the negative samples are uniformly distributed over sequence lengths and the distribution is significantly different from that of the positive peptides. In addition, the dataset is highly unbalanced with significantly more negative samples. Given the different distributions between the IEDB dataset and the Mass Spectrometry benchmark dataset, models trained using IEDB data without altering its negative sample distribution will not work well on the Mass Spectrometry benchmark dataset.

Even though the Mass Spectrometry benchmark dataset has a data distribution that is different from IEDB, it is still valid to be used as an independent test set. However, to ensure it is truly “independent”, its label information including the distributions of positive and negative samples must not be available or used during the model training process. If a method does the same negative sampling and constructs a similar training data distribution as in the benchmark dataset during its training process, implicitly it uses the label information from test set. Thus, such training process violates the “independence” of the test set and the model performance could be over-estimated. We noticed that MHCflurry uses the same negative sampling method as in the Mass Spectrometry benchmark dataset during its training process, which might not be appreciate. In Tables 7 and 8, we still used the same negative sampling method as what MHCflurry used in our ensemble methods just to make a fair comparison with MHCflurry.

### 8.2 Reproducibility

We published our data and code at https://github.com/ziqi92/peptide-binding-prediction.

## 9 Conclusion

Our methods contribute to the study of peptide-MHC binding prediction problem in two ways. First, instead of predicting the exact binding affinities values as in the existing methods, we formulate the problem as to prioritize most possible peptide-MHC binding pairs via a ranking-based learning. We developed three pairwise ranking-based learning objectives for such prioritization, and the corresponding learning methods that impose the peptide-MHC pairs of higher binding affinities ranked above those with lower binding affinities with a certain margin. Our experimental results in comparison with the state-of-the-art regression based methods demonstrate the superior prediction performance of our methods in prioritizing and identifying the most likely binding peptides. In addition to the learning objectives, we also developed two convolutional neural network-based model architectures ConvM and SpConvM, which incorporate a new position encoding method and attention mechanism that differentiate the importance of amino acids at different positions in determining peptide-MHC binding. Our experiments show that the learned important positions and amino acids for allele HLA-A-0201 conform to the biological understanding of the allele. Our experimental results also demonstrate that our model architectures can achieve superior or at least comparable performance with the state-of-the-art allele-specific baseline MHCflurry.

## Data Availability

Publicly available datasets were analyzed in this study. This data can be found here: The Immune Epitope Database (IEDB) https://www.iedb.org/home_v3.php.
