# Supplementary material for: Ranking-Based Convolutional Neural Network Models for Peptide-MHC Class I Binding Prediction
Source: Front Mol Biosci. 2021 May 17;8:634836. doi: 10.3389/fmolb.2021.634836 (PMC8165219; doi:10.3389/fmolb.2021.634836)
Supplement: Supplementary file 1 [file Presentation1.pdf]

Ranking-based Convolutional Neural Network Models for Peptide-MHC Class I Binding Prediction (Supplementary Materials)

Ziqi Chen [1], **Martin Renqiang Min** [2] **and Xia Ning** [1,3,4]

[1] *Computer Science and Engineering Department, The Ohio State University, Columbus, OH, US*
[2] *Machine Learning Department, NEC Labs America, Princeton, New Jersey, NJ, US*
[3] *Biomedical Informatics Department, The Ohio State University, Columbus, OH, US*
[4] *Translational Data Analytics Institute, The Ohio State University, Columbus, OH, US*

Correspondence*:
Xia Ning
ning.104@osu.edu

APPENDIX

A1 ADDITIONAL EXPERIMENTAL RESULTS

A1.1 Hyperparameter Search Space

The search space of hyperparameters includes batch size {32, 128}, number of layers {1, 2}, filter size {1, 3, 5}, number of filters {8, 16, 64} and the size of output layer {8, 16, 64}.

A1.2 Model Architecture Comparison

Table A1, A2, A3 and A4 present the average percentage improvement of our models over the baseline `MHCflurry` with MS. Models are trained with `BLOSUM` +`Onehot` +`Deep` and selected with respect to 4 different metrics (i.e., $\texttt{AR}_{100}$, $\texttt{HR}_{100}$, `AUC` and $\texttt{ROC}_{5}$, respectively).

A1.3 Encoding Method Comparison

Table A5, A6, A7, and A8 present the average percentage improvement of the 7 encoding methods over the baseline encoding method `BLOSUM`. We used `SpConvM` with H_v as the model to evaluate encoding methods and their combination. We trained the models and selected the hyper-parameters with respect to 4 different metrics (i.e., $\texttt{AR}_{100}$, $\texttt{HR}_{100}$, `AUC` and $\texttt{ROC}_{5}$, respectively).

Table A1. Performance Comparison over MHCflurry with MS (AR_{100}; BLOSUM+Onehot+Deep)

Model	loss	AR_{100}	HR_{100}	AR_{500}	HR_{500}	AUC	ROC_5	ROC_{10}
ConvM	H_v	8.08	4.79	3.56	5.93	6.36	9.92	10.01
	H_l	6.36	4.50	1.33	4.50	5.21	6.57	5.92
	H_i	6.58	3.49	1.36	3.26	5.63	7.25	6.05
	MS	-5.17	-7.93	-6.69	-1.93	1.24	-3.51	-3.36
SpConvM	H_v	10.75	7.58	**8.06**	**9.61**	**8.68**	**18.84**	**16.29**
	H_l	10.10	5.39	5.87	6.69	7.39	14.09	10.97
	H_i	10.59	**8.28**	5.32	7.47	7.35	14.66	11.56
	MS	7.36	7.54	3.27	5.74	5.16	15.66	11.68
MHCflurry	H_v	**10.95**	6.74	6.62	7.95	6.45	15.87	11.45
	H_l	8.61	7.32	5.37	6.08	5.71	10.75	9.39
	H_i	8.20	4.19	4.87	5.87	4.92	11.79	7.99
	MS	0.00	0.00	0.00	0.00	0.00	0.00	0.00

The values in the table are percentage improvement compared with the baseline MHCflurry with MS. Models are trained using BLOSUM+Onehot+Deep encoding methods, and selected with respect to AR_{100} and evaluated using the 7 evaluation metrics. The best improvement with respect to each metric is **bold**.

Table A2. Performance Comparison over MHCflurry with MS (HR_{100}; BLOSUM+Onehot+Deep)

Model	loss	AR_{100}	HR_{100}	AR_{500}	HR_{500}	AUC	ROC_5	ROC_{10}
ConvM	H_v	5.72	6.89	3.15	5.66	7.11	12.78	10.81
	H_l	4.89	5.54	2.47	3.50	6.27	7.34	8.35
	H_i	4.75	5.88	0.93	3.62	6.24	7.21	7.29
	MS	-8.57	0.21	-8.00	-1.23	1.65	-2.45	-2.47
SpConvM	H_v	9.86	**8.85**	**7.89**	8.59	**9.12**	**19.29**	**18.11**
	H_l	8.18	7.64	6.46	6.64	8.44	15.59	14.08
	H_i	7.35	8.5	5.02	6.41	7.96	13.21	14.04
	MS	7.10	8.05	2.49	4.95	6.30	15.17	12.5
MHCflurry	H_v	**10.11**	8.54	7.14	**9.21**	6.09	16.00	14.65
	H_l	8.78	6.60	5.55	5.95	5.50	13.19	11.30
	H_i	6.50	6.20	5.08	5.47	4.69	12.19	8.12
	MS	0.00	0.00	0.00	0.00	0.00	0.00	0.00

The values in the table are percentage improvement compared with the baseline MHCflurry with MS. Models are trained using BLOSUM+Onehot+Deep encoding methods, and selected with respect to HR_{100} and evaluated using the 7 evaluation metrics. The best improvement with respect to each metric is **bold**.

Table A3. Performance Comparison over `MHCflurry` with MS (`AUC`; `BLOSUM+Onehot+Deep`)

Model	loss	AR_{100}	HR_{100}	AR_{500}	HR_{500}	AUC	ROC_5	ROC_{10}
ConvM	H_v	6.83	2.28	5.03	7.34	3.44	9.69	8.55
	H_l	3.00	3.12	3.88	6.88	3.16	5.41	5.81
	H_i	3.66	-0.63	3.01	5.83	2.81	4.43	3.58
	MS	-9.12	-8.45	-5.46	-2.31	-0.52	-5.62	-5.45
SpConvM	H_v	**10.27**	**4.42**	**9.24**	**10.08**	**5.09**	**17.20**	**15.06**
	H_l	7.18	2.84	8.12	8.31	4.42	12.36	10.63
	H_i	7.54	2.98	7.69	7.25	4.44	11.57	10.04
	MS	4.31	2.49	5.28	4.78	2.86	11.68	9.47
MHCflurry	H_v	8.08	1.61	7.45	8.35	4.30	11.02	11.05
	H_l	7.64	3.96	6.31	7.61	3.56	8.63	7.63
	H_i	7.39	1.64	5.81	6.74	3.41	9.32	7.68
	MS	0.00	0.00	0.00	0.00	0.00	0.00	0.00

The values in the table are percentage improvement compared with the baseline `MHCflurry` with MS. Models are trained using `BLOSUM+Onehot+Deep` encoding methods, and selected with respect to `AUC` and evaluated using the 7 evaluation metrics. The best improvement with respect to each metric is **bold**.

Table A4. Performance Comparison over `MHCflurry` with MS (ROC_5; `BLOSUM+Onehot+Deep`)

Model	loss	AR_{100}	HR_{100}	AR_{500}	HR_{500}	AUC	ROC_5	ROC_{10}
ConvM	H_v	8.38	6.71	3.23	6.86	5.14	5.91	4.63
	H_l	3.33	5.16	0.96	5.52	4.22	5.22	2.56
	H_i	8.36	7.95	3.35	5.83	5.01	5.40	3.70
	MS	-11.59	-5.75	-8.19	-2.14	0.23	-7.20	-8.84
SpConvM	H_v	**12.78**	**15.81**	**8.31**	**11.11**	**7.29**	**15.03**	**10.52**
	H_l	11.71	9.88	5.45	8.12	6.20	12.09	7.72
	H_i	10.18	8.09	7.40	9.58	6.65	10.38	8.09
	MS	5.25	3.68	2.96	4.81	4.49	13.54	6.87
MHCflurry	H_v	11.20	11.53	7.09	7.44	6.32	10.38	8.31
	H_l	1.87	0.36	0.09	1.16	3.22	13.76	9.80
	H_i	7.64	8.25	5.83	6.28	5.36	8.66	7.74
	MS	0.00	0.00	0.00	0.00	0.00	0.00	0.00

The values in the table are percentage improvement compared with the baseline `MHCflurry` with MS. Models are trained using `BLOSUM+Onehot+Deep` encoding methods, and selected with respect to ROC_5 and evaluated using the 7 evaluation metrics. The best improvement with respect to each metric is **bold**.

Table A5. Encoding Performance Comparison on `SpConvM` with H_v (AR_{100})

encoding	AR_{100}	HR_{100}	AR_{500}	HR_{500}	AUC	ROC_5	ROC_{10}
BLOSUM	0.00	0.00	0.00	0.00	0.00	0.00	0.00
Onehot	-6.04	-4.09	-6.00	-1.75	-1.13	-5.38	-5.34
Deep	-4.74	-0.98	-2.55	-0.09	-0.13	0.26	-1.17
BLOSUM +Onehot	-0.62	-0.06	-0.62	1.68	0.27	3.17	1.22
BLOSUM +Deep	**1.43**	**3.33**	**0.66**	2.62	**0.57**	4.88	2.73
Onehot +Deep	-4.10	-3.16	-2.64	-0.37	0.01	-1.83	-2.29
BLOSUM +Onehot +Deep	0.15	-0.15	-0.47	**3.53**	0.53	**6.29**	**3.53**

The values in the table are percentage improvement compared with the `BLOSUM` encoding method. Models are selected with respect to AR_{100} and evaluated using the 7 evaluation metrics. The best improvement with respect to each metric is **bold**.

Table A6. Encoding Performance Comparison on SpConvM with H_v (HR_{100})

encoding	AR_{100}	HR_{100}	AR_{500}	HR_{500}	AUC	ROC_5	ROC_{10}
BLOSUM	0.00	0.00	0.00	0.00	0.00	0.00	0.00
Onehot	-6.20	-3.31	-5.71	-3.85	-0.69	-7.10	-5.47
Deep	-3.81	-0.44	-2.64	-0.21	0.26	-0.36	0.09
BLOSUM +Onehot	-1.47	0.98	-0.70	-0.04	0.11	1.61	0.85
BLOSUM +Deep	-0.40	**2.45**	-0.30	0.57	0.28	3.39	1.97
Onehot +Deep	-6.45	-2.48	-4.34	-1.71	-0.55	-3.08	-3.13
BLOSUM +Onehot +Deep	**0.58**	0.07	**0.98**	**2.16**	**0.75**	**4.56**	**3.50**

The values in the table are percentage improvement compared with the BLOSUM encoding method. Models are selected with respect to HR_{100} and evaluated using the 7 evaluation metrics. The best improvement with respect to each metric is **bold**.

Table A7. Encoding Performance Comparison on SpConvM with H_v (AUC)

encoding	AR_{100}	HR_{100}	AR_{500}	HR_{500}	AUC	ROC_5	ROC_{10}
BLOSUM	0.00	0.00	0.00	0.00	0.00	0.00	0.00
Onehot	-6.75	-4.84	-4.37	-2.55	-0.91	-6.85	-5.34
Deep	-3.31	-0.59	-2.09	-0.71	-0.19	-1.06	-1.49
BLOSUM +Onehot	-0.45	**0.97**	0.22	0.52	0.09	1.41	1.02
BLOSUM +Deep	**0.95**	-0.84	1.09	**1.58**	0.55	3.25	2.00
Onehot +Deep	-2.90	-3.40	-2.68	-1.56	-0.39	-4.13	-3.26
BLOSUM +Onehot +Deep	0.77	-0.67	**1.43**	1.05	**0.62**	**5.90**	**3.95**

The values in the table are percentage improvement compared with the BLOSUM encoding method. Models are selected with respect to AUC and evaluated using the 7 evaluation metrics. The best improvement with respect to each metric is **bold**.

Table A8. Encoding Performance Comparison on SpConvM with H_v (ROC_5)

encoding	AR_{100}	HR_{100}	AR_{500}	HR_{500}	AUC	ROC_5	ROC_{10}
BLOSUM	0.00	0.00	0.00	0.00	0.00	0.00	0.00
Onehot	-7.96	-7.56	-5.22	-2.32	-1.05	-5.61	-4.81
Deep	-2.29	-0.84	-2.76	-0.78	-0.54	-0.69	-1.28
BLOSUM +Onehot	1.24	-0.16	0.18	0.40	0.00	1.38	1.12
BLOSUM +Deep	**2.84**	0.15	1.41	**1.90**	0.60	3.20	3.14
Onehot +Deep	-4.76	-2.90	-3.31	-1.28	-0.61	-2.58	-2.30
BLOSUM +Onehot +Deep	2.57	**0.20**	**2.08**	1.46	**0.82**	**3.83**	**3.47**

The values in the table are percentage improvement compared with the BLOSUM encoding method. Models are selected with respect to ROC_5 and evaluated using the 7 evaluation metrics. The best improvement with respect to each metric is **bold**.
